# Supplementary figures and images for: High activity of an affinity-matured ACE2 decoy against Omicron SARS-CoV-2 and pre-emergent coronaviruses
Source: PLoS One. 2022 Aug 25;17(8):e0271359. doi: 10.1371/journal.pone.0271359 (PMC9409550; doi:10.1371/journal.pone.0271359)

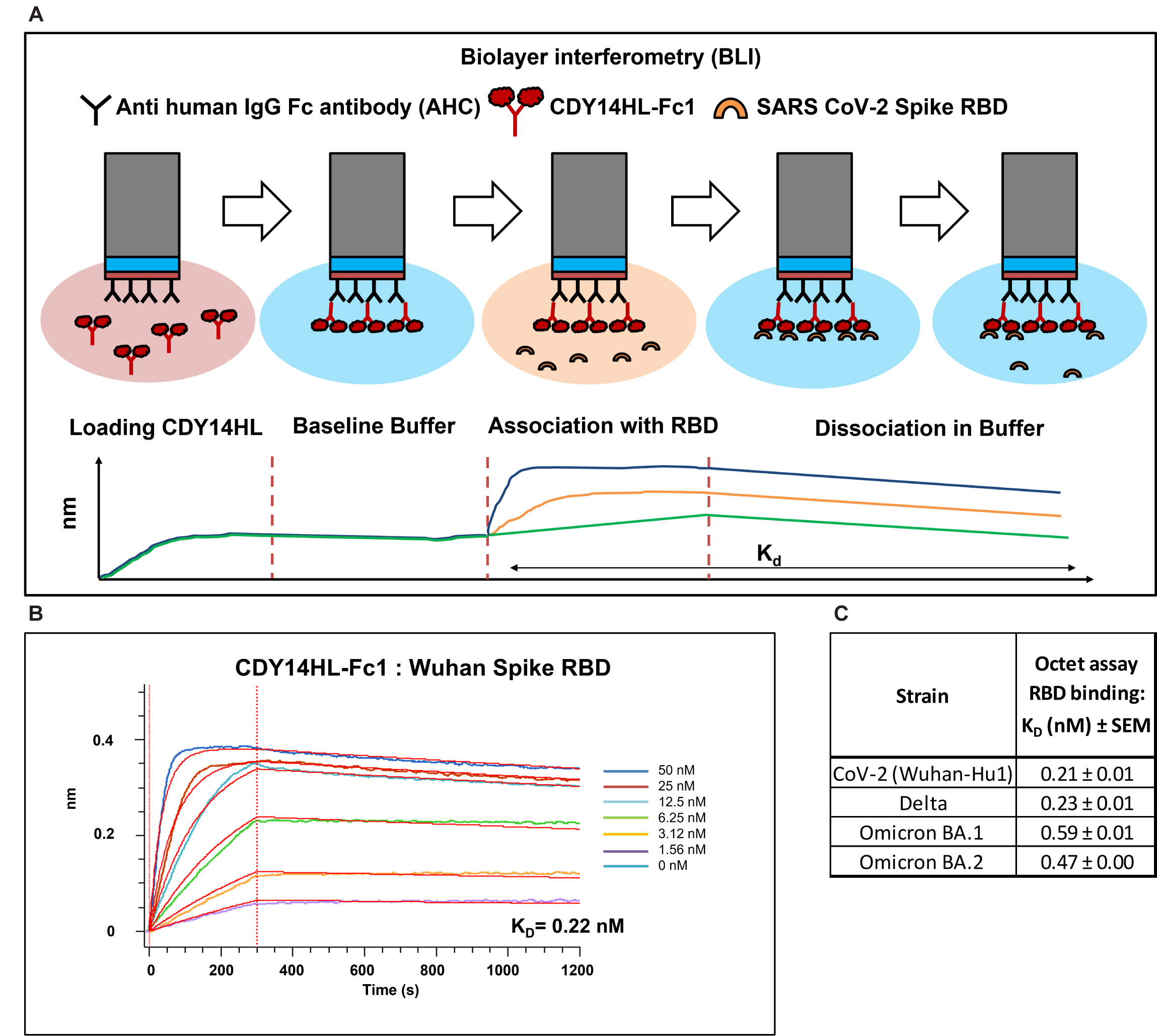

Supplement: S1 Fig — A) Depiction of Biolayer interferometry assay format. CDY14HL-Fc1 was immobilized as ligand and SARS-CoV-2 variant RBD was used as analyte. B) Representative fitted sensogram for CDY14HL-Fc1 and CoV-2 Wuhan-Hu-1 spike RBD. C) Table of KD values (nM) for CDY14HL-Fc1 and four SARS-CoV-2 variant RBDs. Mean values and standard error of the mean (SEM) determined from three technical replicates. (TIF) [file pone.0271359.s001.tif]

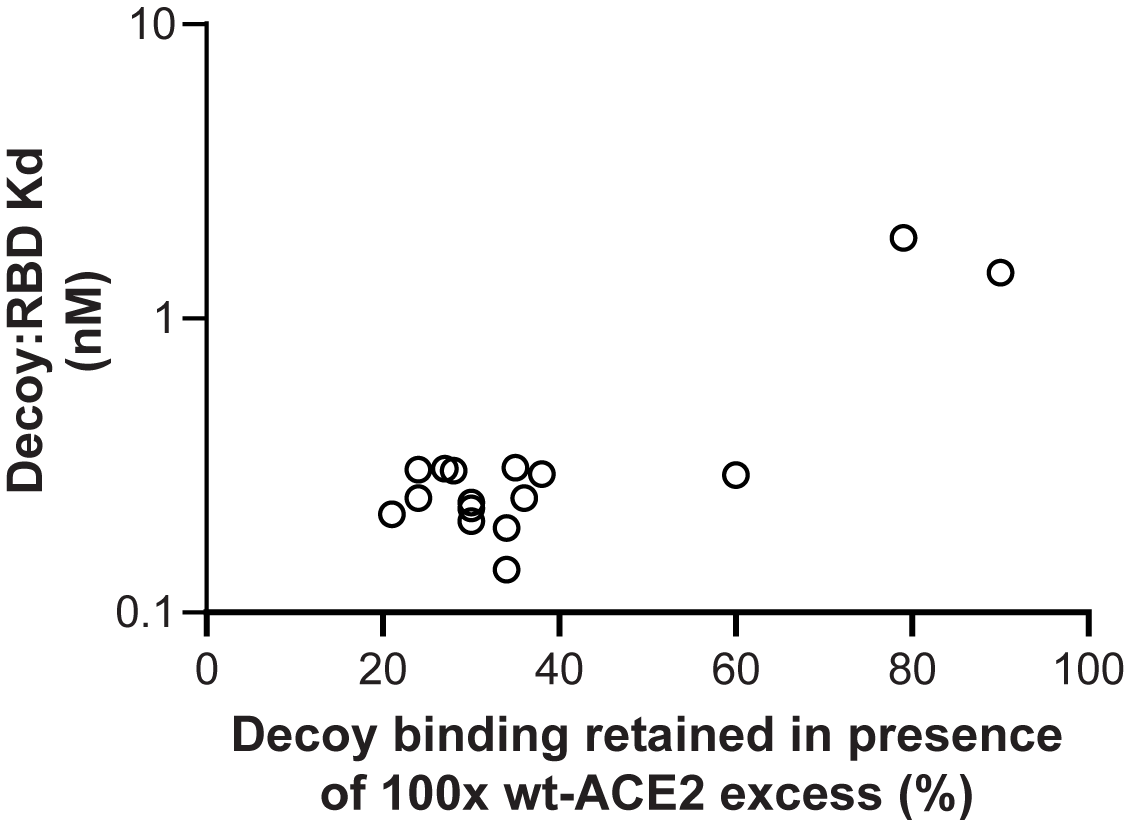

Supplement: S2 Fig — (TIF) [file pone.0271359.s002.tif]
