# Supplementary material for: High activity of an affinity-matured ACE2 decoy against Omicron SARS-CoV-2 and pre-emergent coronaviruses
Source: PLoS One. 2022 Aug 25;17(8):e0271359. doi: 10.1371/journal.pone.0271359 (PMC9409550; doi:10.1371/journal.pone.0271359)
Supplement: S1 File — (DOCX) [file pone.0271359.s003.docx]

**Supplementary Information**

**Materials and Methods**

**Biolayer interferometry**

For the determination of binding affinity, biolayer interferometry measurements were taken on an Octet HTX (Sartorius Corporation). Purified CDY14HL-Fc1 was immobilized on an anti-human IgG Fc sensor (AHC sensor, Sartorius Corporation). Decoy loaded tips were dipped into wells containing purified His-tagged SARS-CoV-2 Spike RBD proteins (R&D Biotechne Corporation). RBD concentrations of 50, 25, 12.5, 6,25, 3.125 and 1.56 nM were used to determine the K_D_s. The diluent and running buffer for all experiments was 1x Kinetics Buffer (FortéBio, PBS+ 0.02% Tween20, 0.1% BSA, 0.05% sodium azide). K_D_ fits were determined by 1:1 binding model global grouped fits on the Octet Data Analysis HT Version 12.0.2.59 (FortéBio). All measurements were conducted in triplicate.

**Plasmid and sequence details for constructs used in the main text**

Nucleotide sequence of CDY14HL-Fc1:

ATGTCAAGCTCTTCCTGGCTCCTTCTCAGCCTTGTTGCTGTAACTGCTGCTCAGTCCACCATTGAGGAACAGGCCAAGACATTTTTGGACAtGTTTAACCACaAAGCCGAAGACCTGTTCTATCAAAGTTCACTTGCTgCTTGGAATTATAACACCAATATTACTGAAGAGAATGTCCAAAACATGAATAAcGCTGGGGACAAATGGTCTGCCTTTTTAAAGGAACAGTCCACAtTTGCCCAAATGTATCCACTACAAGAAATTCAGAATCcCACAGTCAAGCTTCAGCTGCAGGCTCTTCAGCAAAATGGGTCTTCAGTGCTCTCAGAAGACAAGAGCAAACGGTTGAACACAATTCTAAATACAATGAGCACCATCTACAGTACTGGAAAAGTTTGTAACCCgGATAATCCACAAGAATGCTTATTACTTGAACCAGGTTTGAATGAAATAATGGCAAACAGTTTAGACTACAATGAGAGGCTCTGGGCTTGGGAAAGCTGGAGATCTGAGGTCGGCAAGCAGCTGAGGCCATTATATGAAGAGTATGTGGTCTTGAAAAATGAGATGGCgAGAGCAAAcCATTATGAGGACTATGGGGATTATTGGAGAGGAGACTATGAAGTAAATGGGGTAGATGGCTATGACTACAGCCGCGGCCAGTTGATTGAAGATGTGGAACATACCTTTGAAGAGATTAAACCATTATATGAACATCTTCATGCCTATGTGAGGGCAAAGTTGATGAATGCCTATCCTTCCTATATCAGTCCAATTGGATGCCTCCCTGCTCATTTGCTTGGTGATATGTGGGGTAGATTTTGGACAAATCTGTACTCTTTGACAGTTCCCTTcGGACAGAAACCAAACATAGATGTTACTGATGCAATGGTGGACCAGGCCTGGGATGCACAGAGAATATTCAAGGAGGCCGAGAAGTTCTTTGTATCTGTTGGTCTTCCTAATATGACTCAAGGATTCTGGGAAtATTCCATGCTAACGGACCCAGGAAATGTTCAGAAAGCAGTCTGCCtTCCCACAGCTTGGGACCTGGGGAAGGGCGACTTCAGGATCCTTATGTGCACAAAGGTGACAATGGACGACTTCCTGACAGCTCATCATGAGATGGGGCATATCCAGTATGATATGGCATATGCTGCACAACCTTTTCTGCTAAGAAATGGAGCTAATGAAGGATTCCATGAAGCTGTTGGGGAAATCATGTCACTTTCTGCAGCCACACCTAAGCATTTAAAATCCATTGGTCTTCTGTCACCCGATTTTCAAGAAGACAATGAAACAGAAATAAACTTCCTGCTCAAACAAGCACTCACGATTGTTGGGACTCTGCCATTTACTTACATGTTAGAGAAGTGGAGGTGGATGGTCTTTAAAGGGGAAATTCCCAAAGACCAGTGGATGAAAAAGTGGTGGGAGATGAAGCGAGAGATAGTTGGGGTGGTGGAACCTGTGCCCCATGATGAAACATACTGTGACCCCGCATCTCTGTTCCATGTTTCTAATGATTACTCATTCATTCGATATTACACAAGGACCCTTTACCAATTCCAGTTTCAAGAAGCACTTTGTCAAGCAGCTAAACATGAAGGCCCTCTGCACAAATGTGACATCTCAAACTCTACAGAAGCTGGACAGAAACTGTTCAATATGCTGAGGCTTGGAAAATCAGAACCCTGGACCCTAGCATTGGAAAATGTTGTAGGAGCAAAGAACATGAATGTAAGGCCACTGCTCAACTACTTTGAGCCCTTATTTACCTGGCTGAAAGACCAGAACAAGAATTCTTTTGTGGGATGGAGTACCGACTGGAGTCCATATGCAGACggaagtGACAAGACCCACACCTGTCCTCCATGTCCTGCTCCAGAACTGCTCGGCGGACCTTCCGTGTTCCTGTTTCCTCCAAAGCCTAAGGACACCCTGATGATCAGCAGAACCCCTGAAGTGACCTGCGTGGTGGTGGATGTGTCCCACGAGGATCCCGAAGTGAAGTTCAATTGGTACGTGGACGGCGTGGAAGTGCACAACGCCAAGACCAAGCCTAGAGAGGAACAGTACAACAGCACCTACAGAGTGGTGTCCGTGCTGACCGTGCTGCACCAGGATTGGCTGAACGGCAAAGAGTACAAGTGCAAGGTGTCCAACAAGGCCCTGCCTGCTCCTATCGAGAAAACCATCAGCAAGGCCAAGGGCCAGCCTAGGGAACCCCAGGTTTACACACTGCCTCCAAGCAGGGACGAGCTGACCAAGAATCAGGTGTCCCTGACCTGCCTCGTGAAGGGCTTCTACCCTTCCGATATCGCCGTGGAATGGGAGAGCAACGGCCAGCCTGAGAACAACTACAAGACAACCCCTCCTGTGCTGGACAGCGACGGCTCATTCTTCCTGTACAGCAAGCTGACAGTGGACAAGTCCAGGTGGCAGCAGGGCAACGTGTTCAGCTGCAGCGTGATGCACGAGGCCCTGCACAACCACTACACCCAGAAGTCCCTGAGCCTGTCTCCTGGAaaaTGATGA

Nucleotide sequence of wt-ACE2-mFc (human ACE2 1-615 fused to Fc from mouse IgG2A)

ATGTCAAGCTCTTCCTGGCTCCTTCTCAGCCTTGTTGCTGTAACTGCTGCTCAGTCCACCATTGAGGAACAGGCCAAGACATTTTTGGACAAGTTTAACCACGAAGCCGAAGACCTGTTCTATCAAAGTTCACTTGCTTCTTGGAATTATAACACCAATATTACTGAAGAGAATGTCCAAAACATGAATAATGCTGGGGACAAATGGTCTGCCTTTTTAAAGGAACAGTCCACACTTGCCCAAATGTATCCACTACAAGAAATTCAGAATCTCACAGTCAAGCTTCAGCTGCAGGCTCTTCAGCAAAATGGGTCTTCAGTGCTCTCAGAAGACAAGAGCAAACGGTTGAACACAATTCTAAATACAATGAGCACCATCTACAGTACTGGAAAAGTTTGTAACCCAGATAATCCACAAGAATGCTTATTACTTGAACCAGGTTTGAATGAAATAATGGCAAACAGTTTAGACTACAATGAGAGGCTCTGGGCTTGGGAAAGCTGGAGATCTGAGGTCGGCAAGCAGCTGAGGCCATTATATGAAGAGTATGTGGTCTTGAAAAATGAGATGGCAAGAGCAAATCATTATGAGGACTATGGGGATTATTGGAGAGGAGACTATGAAGTAAATGGGGTAGATGGCTATGACTACAGCCGCGGCCAGTTGATTGAAGATGTGGAACATACCTTTGAAGAGATTAAACCATTATATGAACATCTTCATGCCTATGTGAGGGCAAAGTTGATGAATGCCTATCCTTCCTATATCAGTCCAATTGGATGCCTCCCTGCTCATTTGCTTGGTGATATGTGGGGTAGATTTTGGACAAATCTGTACTCTTTGACAGTTCCCTTTGGACAGAAACCAAACATAGATGTTACTGATGCAATGGTGGACCAGGCCTGGGATGCACAGAGAATATTCAAGGAGGCCGAGAAGTTCTTTGTATCTGTTGGTCTTCCTAATATGACTCAAGGATTCTGGGAAAATTCCATGCTAACGGACCCAGGAAATGTTCAGAAAGCAGTCTGCCATCCCACAGCTTGGGACCTGGGGAAGGGCGACTTCAGGATCCTTATGTGCACAAAGGTGACAATGGACGACTTCCTGACAGCTCATCATGAGATGGGGCATATCCAGTATGATATGGCATATGCTGCACAACCTTTTCTGCTAAGAAATGGAGCTAATGAAGGATTCCATGAAGCTGTTGGGGAAATCATGTCACTTTCTGCAGCCACACCTAAGCATTTAAAATCCATTGGTCTTCTGTCACCCGATTTTCAAGAAGACAATGAAACAGAAATAAACTTCCTGCTCAAACAAGCACTCACGATTGTTGGGACTCTGCCATTTACTTACATGTTAGAGAAGTGGAGGTGGATGGTCTTTAAAGGGGAAATTCCCAAAGACCAGTGGATGAAAAAGTGGTGGGAGATGAAGCGAGAGATAGTTGGGGTGGTGGAACCTGTGCCCCATGATGAAACATACTGTGACCCCGCATCTCTGTTCCATGTTTCTAATGATTACTCATTCATTCGATATTACACAAGGACCCTTTACCAATTCCAGTTTCAAGAAGCACTTTGTCAAGCAGCTAAACATGAAGGCCCTCTGCACAAATGTGACATCTCAAACTCTACAGAAGCTGGACAGAAACTGTTCAATATGCTGAGGCTTGGAAAATCAGAACCCTGGACCCTAGCATTGGAAAATGTTGTAGGAGCAAAGAACATGAATGTAAGGCCACTGCTCAACTACTTTGAGCCCTTATTTACCTGGCTGAAAGACCAGAACAAGAATTCTTTTGTGGGATGGAGTACCGACTGGAGTCCATATGCAGACCCCAGAGGGCCCACAATCAAGCCCTGTCCTCCATGCAAATGCCCAGCACCTAACCTCTTGGGTGGACCATCCGTCTTCATCTTCCCTCCAAAGATCAAGGATGTACTCATGATCTCCCTGAGCCCCATAGTCACATGTGTGGTGGTGGATGTGAGCGAGGATGACCCAGATGTCCAGATCAGCTGGTTTGTGAACAACGTGGAAGTACACACAGCTCAGACACAAACCCATAGAGAGGATTACAACAGTACTCTCCGGGTGGTCAGTGCCCTCCCCATCCAGCACCAGGACTGGATGAGTGGCAAGGAGTTCAAATGCAAGGTCAACAACAAAGACCTCCCAGCGCCCATCGAGAGAACCATCTCAAAACCCAAAGGGTCAGTAAGAGCTCCACAGGTATATGTCTTGCCTCCACCAGAAGAAGAGATGACTAAGAAACAGGTCACTCTGACCTGCATGGTCACAGACTTCATGCCTGAAGACATTTACGTGGAGTGGACCAACAACGGGAAAACAGAGCTAAACTACAAGAACACTGAACCAGTCCTGGACTCTGATGGTTCTTACTTCATGTACAGCAAGCTGAGAGTGGAAAAGAAGAACTGGGTGGAAAGAAATAGCTACTCCTGTTCAGTGGTCCACGAGGGTCTGCACAATCACCACACGACTAAGAGCTTCTCCCGGACTCCGGGTAAATGA

**Map of typical Aga2-RBD-HA construct used for yeast display**


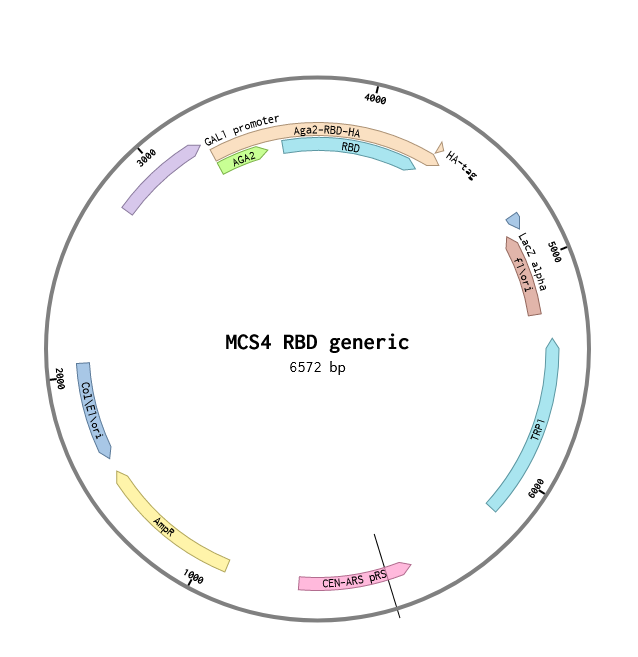


Sequence of Aga2-Wuhan-Hu-1-RBD-HA

ATGCAGTTACTTCGCTGTTTTTCAATATTTTCTGTTATTGCTTCAGTTTTAGCACAGGAACTGACAACTATATGCGAGCAAATCCCCTCACCAACTTTAGAATCGACGCCGTACTCTTTGTCAACGACTACTATTTTGGCCAACGGGAAGGCAATGCAAGGAGTTTTTGAATATTACAAATCAGTAACGTTTGTCAGTAATTGCGGTTCTCACCCCTCAACAACTAGCAAAGGCAGCCCCATAAACACACAGTATGTTTTTAAGGACAATAGCTCGACGATTGAAGGTAGAGGATCCGGAGGTAGCGGATCAGGAGGTGGAGGCTCCGGAGGAGGTGGACCAAACATCACCAATCTGTGCCCCTTCGGCGAAGTTTTCAACGCCACCAGATTCGCCAGCGTGTACGCCTGGAATAGAAAGCGGATCAGCAACTGTGTGGCCGACTACAGCGTGCTGTATAACAGCGCCAGCTTCAGCACATTCAAGTGCTACGGCGTGTCCCCTACCAAGCTGAACGACCTGTGTTTTACCAACGTGTACGCCGATAGCTTCGTGATTAGAGGCGACGAGGTGCGGCAGATCGCCCCTGGCCAGACCGGTAAGATCGCTGATTACAACTACAAGCTGCCTGACGACTTCACCGGCTGTGTGATCGCCTGGAACAGCAACAACCTGGACAGCAAGGTGGGCGGCAACTATAACTACCTGTACCGGCTGTTCAGAAAATCTAATCTGAAGCCCTTCGAGAGAGATATCAGCACAGAGATCTACCAGGCCGGCAGCACCCCTTGCAACGGCGTGGAAGGCTTTAACTGCTACTTCCCCCTGCAGAGCTACGGATTCCAGCCAACAAATGGAGTCGGCTACCAACCTTACAGAGTGGTCGTGCTCTCCTTTGAGCTGCTGCACGCCCCTGCCACAGTGTGCGGACCTAAGAAAAGCGGATCTTCTGGATCTGGATCTGGATCTGAATCTAAGTCTACTGGATACCCATACGACGTTCCAGACTACGCTCTGCAGGCTAGTGGTTGA
